# Mechanical Elongation of the Small Intestine: Evaluation of Techniques for Optimal Screw Placement in a Rodent Model

**DOI:** 10.1155/2013/601701

**Published:** 2013-07-24

**Authors:** P. A. Hausbrandt, H. Ainoedhofer, A. K. Saxena, J. Schalamon

**Affiliations:** Department of Pediatric and Adolescent Surgery, Medical University of Graz, Auenbruggerplatz 34, 8036 Graz, Austria

## Abstract

*Introduction*. The aim of this study was to evaluate techniques and establish an optimal method for mechanical elongation of small intestine (MESI) using screws in a rodent model in order to develop a potential therapy for short bowel syndrome (SBS). *Material and Methods*. Adult female Sprague Dawley rats (*n* = 24) with body weight from 250 to 300 g (Σ = 283) were evaluated using 5 different groups in which the basic denominator for the technique involved the fixation of a blind loop of the intestine on the abdominal wall with the placement of a screw in the lumen secured to the abdominal wall. *Results*. In all groups with accessible screws, the rodents removed the implants despite the use of washers or suits to prevent removal. Subcutaneous placement of the screw combined with antibiotic treatment and dietary modifications was finally successful. In two animals autologous transplantation of the lengthened intestinal segment was successful. *Discussion*. While the rodent model may provide useful basic information on mechanical intestinal lengthening, further investigations should be performed in larger animals to make use of the translational nature of MESI in human SBS treatment.

## 1. Introduction

Short bowel syndrome (SBS) is recognized to be a sequel of metabolic and pathophysiological visceral conditions that occur as a result of a remaining small bowel length of less than 30% [1]. However, this definition did not consider the quality of the remaining bowel segments and the individual clinical aspects. SBS is best defined as “intestinal failure resulting from surgical resection, congenital defect or disease associated loss of absorption, characterized by the inability to remain protein-energy, fluid, electrolyte or micro-nutritient [sic] balances when on a conventionally accepted, normal diet” [2]. In the pediatric population SBS results from necrotizing enterocolitis, abdominal wall defects, intestinal atresia, volvulus, or congenital short bowel. Malignancy, radiation, inflammatory bowel disease and vascular insufficiency are the most common causes in adults [3].

The loss of bowel length is compensated by continuous adaption processes of the residual bowel that may lead to a complete functional recovery [4]. This includes structural, physiological, and enzymatic changes like mucosal hyperplasia, increased mucosal blood flow, a higher segmental absorption rate, and hypergastrinic episodes [5]. For this, long term parenteral nutrition is frequently required to restore adequate intake of nutrients but is accompanied by significant complications such as catheter-related sepsis, liver failure, and significant costs up to €80,000–120,000 per patient annually [3]. To improve intestinal function a number of operative procedures have been introduced with limited success: increasing the absorptive intestinal surface [6, 7] and slowing the passage time with segmental reversal operations [8] or intestinal transplantation [9–11]. 

In 1990, the concept of using inflatable silicon balloons similar to tissue expanders used by plastic surgeons for mechanical lengthening was introduced [12]. External fixation devices such as a mechanical distraction device fixed on the outside of the intestine were then reported [13]. The mechanical elongation of small intestine (MESI) using the application of a stainless steel screw was first reported in 2004 and was subsequently applied over the years with modifications and technical failures [14–19]. Another approach for intestinal lengthening was published by Shekherdimian et al. using an endoluminal spring instead of a screw, whereas Stark et al. recently modified this procedure with a self-dissolving capsule [20, 21]. The aim of this study was to evaluate techniques and establish an optimal method for MESI in a rodent model.

## 2. Material and Methods

The experiments were performed after approval from the Animal Ethics Committee, Ministry of Science and Research, Vienna, Austria (GZ 66.010/0058-II/10b/2009). Adult female Sprague Dawley rats (*n* = 24) with body weight 250–300 g (Σ = 283 g) were investigated regarding the feasibility and technical ease/difficulty of MESI using screws as published previously. The procedures were performed under general anaesthesia using Isoflurane while keeping the rodent on a heating plate to maintain normal body temperature. The abdominal skin was shaved and prepped with betadine. For additional perioperative analgesia a subcutaneous mixture of 15 *μ*g/kg Buprenorphine and 5 mg/kg Carprofen was administered. Single dose antibiotic prophylaxis with 7.5 mg/kg Enrofloxacin was also administered. A sagittal midline laparotomy was performed and the caecum localized. In the midsegment of the small intestine (jejunum) a 1.5 cm long vascularised segment was isolated. The continuity of the remaining intestine was restored with an end-end anastomosis. The distal part of the isolated vascularised small intestine was sutured to form a stump. The screws (stainless steel, 4 cm length and 3 mm diameter, purchased in a local tool store, sterilized before usage) were placed according to different techniques (Group 1–5). The screw with its end covered with a silicone cap was advanced 5 mm into the abdominal cavity. The proximal end of the isolated small intestine was sutured to the inner side of the abdominal wall. The screw was turned until minor tension was noticed on the isolated segment. The abdominal wall was sutured. After five days of healing the screws were advanced every third day (five times) until resistance was experienced. 

**Figure 1:**
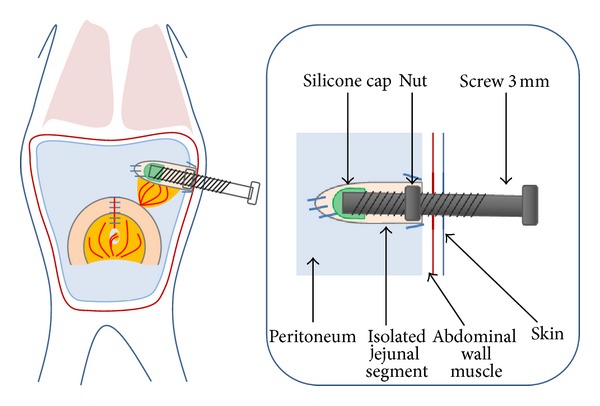
Group 1, direct implantation.

**Figure 2:**
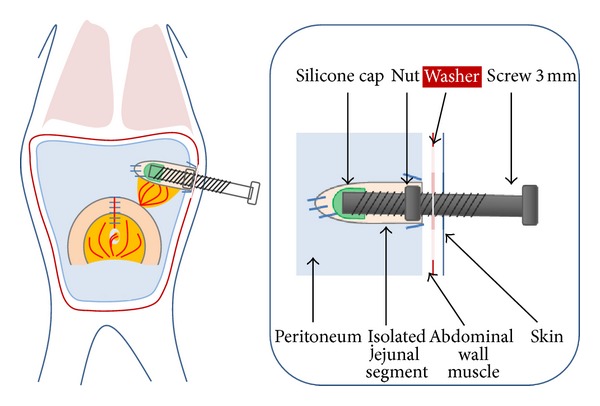
Group 2, submuscular washer and body suit.

**Figure 3:**
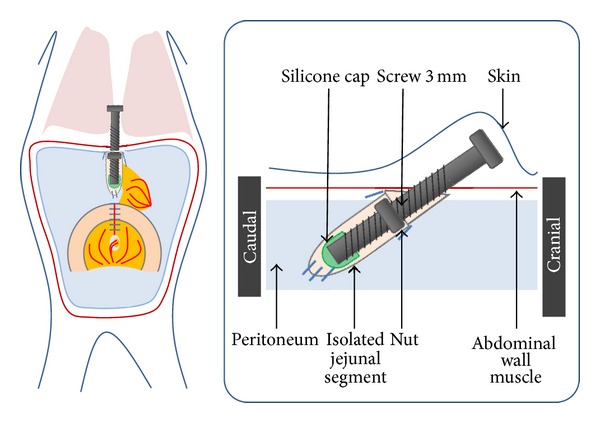
Group 3, subcutaneous screw placement.

*Group 1* (*n* = 8; Figure 1): the screw was introduced through the abdominal wall and anchored with a nut which was placed inside the abdominal wall. We fixed the nut inside the abdominal wall using a purse-string suture to prevent rotation. The outside part of the screw was well accessible to permit advancement. *Group 2* (*n* = 4; Figure 2): to prevent removal of the screw by the rodent, an additional washer was positioned between abdominal wall and nut in order to enlarge the surface of the metal and to provide better fixation in the tissue. Also, the rodent was draped in a gauze suite to cover the outstanding part of the screw. *Group 3* (*n* = 4; Figure 3): the free end of the screw was positioned subcutaneously, to prevent the rodent from manipulating the screw. *Group 4* (*n* = 6): the screw placement was similar to that in *Group 3*, but with changes in the postoperative management with (1) additional analgesics, (2) antibiotic prophylaxis for four days, and (3) feeds of mashed fruits for 1 week (Hipp, Gmunden, Austria). *Group 5* (*n* = 2): the same protocol as *Group 4*, but instead of histological examination the lengthended intestinal segment was replanted to the continuity of an intestinal loop on day 20. The postoperative procedures after transplantation were similar to those in *Group 4*.

## 3. Results 

**Figure 4:**
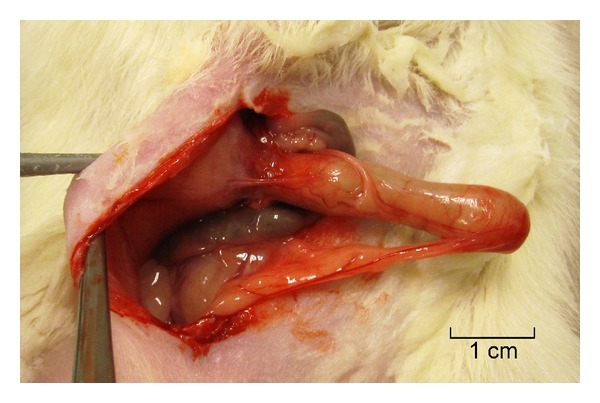
Lengthened intestinal segment.

In *Group 1*, all eight rodents removed the screw in a short period of time, which also included the nut. These animals were euthanized 3–5 days after the primary intervention. In *Group 2*, out of the 4 rodents, all animals managed to slip out of the body suits and mechanically turned the screws to remove them while the nut and the washer remained in place. In *Group 3*, no displacement of the screw was observed. However, the screws placed subcutaneously were associated with postoperative stress, infections, and intestinal obstruction due to adhesions that lead to mortality in all rodents in this group. In *Group 4*, 2 of 6 animals had lethal outcomes on day 11 and day 13 due to adhesive ileus. Intestinal screw implantation was successful with survival in 4 animals. However, the mucus was collected in the lengthened segment and resulted in an increased diameter of the lengthened segment (Figure 4). In *Group 5*, the two rodents did not have complications, the transplantation was well tolerated, and the postoperative course was uneventful.

## 4. Discussion

The idea of tissue expansion is not a new one and is well known in orthopaedic and reconstructive surgery [22, 23]. The elongation of intestines was first performed in 1990 with an inflatable silicon balloon; however, not just the length but also the diameter was increased [12]. The latest development by Shekherdimian et al. was a spring based endoluminal expander device, which was adapted by Stark et al. in 2012 using a self-dissolving capsule to control the expansion of the spring [21, 24]. The reported aim to implant these endoluminal devices endoscopically must be proved in larger animals but from our point of view the occurence of a symptomatic ileus/subileus will be most likely. In our investigations, the method of intestinal lengthening reported by Park et al. was employed, since this method was used successfully in several reports.

However, inability to replicate these results caused modifications of the protocol in our series. Successive modifications of the surgical technique were necessary to achieve proper screw placement with reduction in mortality. Subcutaneous screw placement has been found to be an optimal method, in which self-removal of the screw was not possible. The application of antibiotics and special feeding regimes was associated with better survivability. This surgical modification can be employed for further studies in mechanical bowel lengthening in the rodent model. Successful replantation of the lengthened intestinal segment in the continuity of the small bowel in the rodent model has been reported previously by Stark and coworkers [22]. Further studies are required to evaluate the biological properties of the lengthened segment after successful reintegration. While the rodent model may provide useful basic information on mechanical intestinal lengthening, further investigations should be performed in larger animals in order to make use of the translational nature of MESI in human SBS treatment.
